# Efficient production of recombinant human adiponectin in egg white using genome edited chickens

**DOI:** 10.3389/fnut.2022.1068558

**Published:** 2023-01-24

**Authors:** Young Min Kim, Jin Se Park, Hee Jung Choi, Kyung Min Jung, Kyung Youn Lee, Ji Hyeon Shim, Kyung Je Park, Jae Yong Han

**Affiliations:** ^1^Department of Agricultural Biotechnology and Research Institute of Agriculture and Life Sciences, College of Agriculture and Life Sciences, Seoul National University, Seoul, Republic of Korea; ^2^Avinnogen Co., Ltd., Seoul, Republic of Korea; ^3^Bio-MAX/N-Bio, Institute of BioEngineering, Seoul National University, Seoul, Republic of Korea

**Keywords:** adiponectin, insulin resistance, chicken bioreactor, ovalbumin-targeted gene insertion, fatty acid metabolism

## Abstract

The prevalence of obesity-related metabolic diseases caused by insulin resistance is rapidly increasing worldwide. Adiponectin (ADPN), a hormone derived from adipose tissue, is a potential therapeutic agent for insulin resistance. Chickens are considered efficient bioreactors for recombinant protein production because they secrete large amounts of high-concentration proteins from the oviduct. Additionally, chickens express high levels of high-molecular-weight (HMW) ADPN, which is considered the active form in the body. Therefore, in this study, a gene-targeted chicken model was produced in which the gene encoding human ADPN was inserted into *Ovalbumin* (*OVA)* using the CRISPR/Cas9 system, and the characteristics of the resulting recombinant ADPN protein were evaluated. As a result, human ADPN was expressed in G_1_ hen oviducts and egg whites of *OVA ADPN* knock-in (KI) chickens. The concentration of ADPN in egg white ranged from 1.47 to 4.59 mg/mL, of which HMW ADPN accounted for ∼29% (0.24–1.49 mg/mL). Importantly, egg white-derived ADPN promoted expression of genes related to fatty acid oxidation and activated the 5′-AMP-activated protein kinase (AMPK) signaling pathway in muscle cells. In summary, the *OVA* gene-targeted chicken bioreactor proved to be an advantageous model for production of human ADPN, and the resulting protein was of sufficient quantity and efficacy for industrial use.

## Introduction

Adiponectin (ADPN) is ∼30 kDa protein hormone secreted by adipose tissue (1). In serum, ADPN exists as a low-molecular-weight (LMW) trimer, a middle-molecular-weight (MMW) hexamer, and a high-molecular-weight (HMW) multimer consisting of 12–18 ADPN subunits (2). ADPN levels show a strong negative correlation with body mass index (BMI) in both sexes (3). Furthermore, serum concentrations of ADPN are significantly lower in type 2 diabetes (T2D) patients than in non-diabetic individuals (4). Therefore, serum ADPN levels appear to be correlated with glucose and lipid metabolism. Indeed, ADPN increases insulin sensitivity and has a glucose lowering effect in diabetic mice by inhibiting gluconeogenesis in liver (5). Moreover, ADPN administration stimulates fatty acid oxidation in skeletal muscle, and subsequently reduces triacylglycerol (TAG) and free fatty acid (FFA) levels in tissues and serum, thereby reversing insulin resistance in diabetic mice (6). Other studies showed that ADPN activates 5′-AMP-activated protein kinase (AMPK) in muscle and liver, consequently promoting fatty acid oxidation in muscle and reducing gluconeogenesis in liver (7, 8).

Despite the potential role of ADPN in regulating lipid metabolism, its application for the treatment of human diseases has several limitations. Since ADPN circulates at a relatively high concentration in human serum and has a low half-life, it would be very expensive to use it as a treatment for metabolic diseases (9). In addition, among various oligomeric ADPN forms, the HMW form is considered to have the highest physiological activity in inducing an insulin sensitizing effect and inhibiting hepatic glucose production, but an efficient HMW production method is still not available (10–12). Therefore, an efficient and economical ADPN production system is required for actual clinical use (13).

Chicken eggs are considered as efficient bioreactors because they can produce human proteins with optimal post-transcriptional modifications (PTM) in a cost-effective manner (14). Additionally, chickens are naturally hyperglycemic and considered a good model for insulin resistance (15). It was reported that environmentally high glucose levels can promote multimerization of ADPN, hence, chicken bioreactors may be suitable for the production of multimeric ADPN (16). Interestingly, in chicken, ADPN predominantly exists in the HMW form in serum and tissues, suggesting that the physiological environment of chickens can accelerate multimerization of ADPN (17). Furthermore, chicken oviduct tubular gland cells have a high capacity to produce and secrete proteins into egg white. Ovalbumin (OVA) accounts for ∼54% of total egg white protein, at levels up to 2.2 g per egg (14), indicating that the endogenous OVA promoter is highly active in tubular gland cells. Recent research specifically targeted recombinant protein constructs into OVA loci to express recombinant proteins under the control of the endogenous OVA promoter, and recombinant proteins were successfully accumulated in egg white at levels up to 1 − 4 mg/mL (18, 19). Therefore, chicken bioreactors can serve as an economical production platform for mass-producing recombinant human proteins, and can overcome cost-related issues for the development of human proteins such as ADPN as therapeutic agents. Also, in *ADPN*^±^ mice, levels of the HMW form of ADPN were decreased, which suggests that ADPN expression levels are important for generating the HMW form of ADPN (20). Therefore, when ADPN is expressed under the control of the endogenous OVA promoter, biologically active ADPN can be secreted in high concentrations in the oviduct and accumulate in egg white.

In this study, to produce functional human ADPN economically, we inserted the gene encoding human ADPN into the chicken ovalbumin locus using non-homologous end joining (NHEJ)-mediated knock-in (KI), and produced genome-edited chickens. Through these studies, it can be expected that the chicken bioreactor system can serve as an efficient production platform for functional recombinant human ADPN.

## Materials and methods

### Experimental animals and animal care

The care and experimental use of chickens was approved by the Institute of Laboratory Animal Resources, Seoul National University. Chickens were maintained according to a standard management program at the University Animal Farm, Seoul National University, Korea. All experimental procedures and care of chickens was approved by the Institute of Laboratory Animal Resources, Seoul National University, and all methods were carried out in accordance with ARRIVE (Animal Research: Reporting of *In Vivo* Experiments) guidelines and approved by the Institutional Animal Care and Use Committee (IACUC, SNU-220311-1) of Seoul National University, Korea.

### Construction of chicken *Ovalbumin* gene targeting CRISPR/Cas9 expression plasmids and human adiponectin donor plasmids

The CRISPR/Cas9 vector targeting intron 1 of chicken *OVA* gene was constructed using the PX459 vector (Addgene plasmid #62988). To insert guide RNA (gRNA) sequences into the CRISPR/Cas9 plasmid, sense, and antisense oligonucleotides were designed and synthesized (Bioneer, Daejeon, Korea). These oligonucleotides were annealed under the following thermocycling conditions: 30 s at 95°C, 2 min at 72°C, 2 min at 37°C, and 2 min at 25°C. For targeted gene insertion into the chicken *OVA* gene, a donor cassette containing a part of the intron 1 region, a part of exon 2 including the translation start site of the chicken *Ovalbumin* gene, the human adiponectin gene, and the CMV promoter and puromycin resistance gene was synthesized in the pBHA vector backbone (Bioneer, Daejeon, Korea). The oligonucleotides used for construction of CRISPR/Cas9 vector were listed in Supplementary Table 1.

### Culture of chicken PGC transfection and puromycin-selection of PGCs

The methods of cultivation, transfection and puromycin-selection of WL male PGC cells were followed by our previous report (21). Briefly, male PGCs were cultured on mitotically inactivated mouse fibroblast cells (MEFs) in knockout Dulbecco’s Modified Eagle’s Medium (KO-DMEM) (Invitrogen, Life Technologies, Carlsbad, CA, USA) supplemented with 20% (v/v) fetal bovine serum (Invitrogen, Life Technologies), 2% (v/v) chicken serum (Sigma-Aldrich, St. Louis, MO, USA), 1 × nucleoside mix (EMD Millipore, Temecula, CA, USA), 2 mM L-glutamine, 1 × non-essential amino acid mix, β-mercaptoethanol, 10 mM sodium pyruvate, 1 × antibiotic antimycotic mix (Invitrogen, Life Technologies, Carlsbad, CA, USA), and human basic fibroblast growth factor (10 ng/mL; Koma Biotech, Seoul, Korea). PGCs were cultured at 37°C in an atmosphere of 5% (v/v) CO2 and 60–70% relative humidity. For *OVA* targeted gene insertion in chicken PGCs, donor plasmids (2 μg) and CRISPR/Cas9 expression plasmids (2 μg) were cointroduced into 1 × 10^5^ cultured PGCs with 4 ml of Lipofectamine 2000 (Invitrogen, Life Technologies, Carlsbad, CA, USA) reagent suspended in 1 ml Opti-MEM. Then, 4 h after transfection, the transfection plasmid mixture was replaced with PGC culture medium. One day after transfection, the transfected PGCs were transferred into a new-culture plate with 1 μg/ml puromycin contained culture medium without MEF feeder layer. After selection for 24 h, transfected PGCs were cultured on fresh medium with MEF feeder layer.

### Sequencing analysis of *OVA ADPN* KI PGCs and chickens

For verification of puromycin-selected *OVA ADPN* KI PGCs and *OVA ADPN* KI chickens, PCR was performed using primers specific to 5′ and 3′ junctions for genomic DNA (Supplementary Table 1), and gene insertion was confirmed through sequencing analysis. For sequencing analysis, the amplicons were annealed to the pGEM-T Easy Vector and sequenced using an ABI Prism 3730XLDNAAnalyzer (Thermo Fisher Scientific, Waltham, MA, USA). The sequences were compared against assembled genomes using the Basic Local Alignment Search Tool (BLAST).^1^

### Immunohistochemistry

Chicken oviduct tissues retrieved from WT chickens and *OVA ADPN* KI chickens were paraffin-embedded and sectioned (thickness, 10 μm). After deparaffinization, sections were washed three times with 1 × phosphate-buffered saline (PBS) and blocked with a blocking buffer (5% goat serum and 1% bovine serum albumin in PBS) for 1 h at room temperature. Sections were then incubated at 4°C overnight with a rabbit anti-Adiponectin primary antibody (abcam, Cambridge, UK) (1:200 dilutions in blocking buffer). After washing three times with PBS, sections were incubated with fluorescence-conjugated secondary antibodies (Alexa Fluor 488, Invitrogen) for 1 h at room temperature. After washing three times with PBS, sections were mounted with Prolong Gold antifade reagent with DAPI and imaged using confocal fluorescence microscope (Carl Zeiss GmbH, Oberkocken, Germany).

### SDS PAGE and Western blot analysis

For the analysis of egg white protein, crude egg white was diluted in distilled water and used. Total protein of egg white and of C2C12 cell line was prepared in RIPA lysis buffer (Thermo Fisher Scientific, Waltham, MA, USA) and denatured at 95°C for 5 min in equally mixed with 2 × Laemmli sample buffer (BioRad, CA, USA). For the amount of protein loaded, 1 μg sample was used for SDS PAGE and 25 ng sample was used for Western blotting analysis. The same amount of human recombinant adiponectin was used as a control for the experiments (PEPROTECH, NJ, USA). Then the protein was separated on 10% sodium dodecyl sulfate (SDS)—polyacrylamide gels. The resolved proteins were transferred onto a polyvinylidene fluoride (PVDF) membrane and blocked for 1hr at room temperature. The membrane was incubated with a suitable primary antibody, followed by an appropriate horseradish peroxidase—conjugated secondary antibody (Santa Cruz, TX, USA). The primary antibodies used in this study were as follows: anti-Adiponectin primary antibody (abcam, Cambridge, UK), and AMPK/ACC antibody sampler kit including phospho-AMPKα (Thr172) (40H9), AMPKα (D5A2), phospho-Acetyl-CoA Carboxylase (Ser79), Acetyl-CoA Carboxylase (C83B10), and anti-rabbit IgG, HRP-linked antibody (Cell signaling, MA, USA) used as secondary antibody. Immunoreactive proteins were visualized using the ECL Select Western Blotting Detection Reagent (GE Healthcare Bio-Science, NJ, USA). Signals were detected using a BioRad ChemiDoc XRS imaging system (BioRad, CA, USA).

### Quantification of human adiponectin of *OVA ADPN* egg white

The quantities of total adiponectin and HMW adiponectin from *OVA ADPN* KI egg white were measured using ELISA kits (R&D system) according to the manufacturer’s instructions. In brief, this kit uses the double-antibody sandwich method, the optical density is proportional to the amount of anti-human adiponectin monoclonal antibody present. We measured the concentration of egg derived human adiponectin by comparing the optical density of the standard protein.

### Extraction and purification of chicken adiponectin in transgenic chickens

For chicken Adiponectin purification, the egg-white was added to same volume of 40% ammonium sulfate. The mixture was stirred for overnight at 4°C, and then centrifuged for 1 h at 4°C at 3000 g. The pellet was resuspended in four volumes of 20 mM Tris-HCl, 50 mM NaCl buffer (pH 8.0) to original egg-white volume. Then, the sample was filtered through a 0.22 μm bottle filter. The sample was loaded onto a 5 ml HiTrap Q column (GE Healthcare Bio-Sciences, Uppsala, Sweden), and the protein was eluted with 100 ml gradient 20 mM Tris-HCl, 1 M NaCl buffer (pH 8.0). The protein was further purified and fractionated by size-exclusion chromatography (SEC) using a HiLoad Superdex 75 Column (GE Healthcare Bio-Sciences) pre-equilibrated with 20 mM Tris-HCl, 50 mM NaCl buffer (pH 8.0).

### Quantitative RT-PCR

Total RNA from C2C12 myoblast cell line was extracted using Trizol reagent (Thermo Fisher Scientific, Waltham, MA, USA) and reverse-transcribed using the SuperScript III Reverse Transcription Kit (Thermo Fisher Scientific, Waltham, MA, USA). The complementary DNA (cDNA) was amplified using target gene-specific primer set. Gene expression levels were measured using EvaGreen dye (Biotium, CA, USA) and a CFX96 Real-Time PCR Detection System (BioRad, CA, USA). Each test sample was assayed in triplicate. Quantification of relative gene expression was calculated using following formula: DCt = Ct of the target gene − Ct of *GAPDH*. Primer set information is listed in Supplementary Table 1.

### Statistical analyses

Statistical analysis was performed using GraphPad Prism (GraphPad Software, CA, USA). Significant differences between groups were determined by Student’s *t*-test and one-way ANOVA. A *p*-value < 0.05 was considered statistically significant (^***^p < 0.001, ^**^p < 0.01, and *p < 0.05).

## Results

### Human *ADPN* integrated into the *OVA* locus of primordial germ cells (PGCs) using CRISPR/Cas9, and production of *OVA ADPN* KI chickens

**FIGURE 1:**
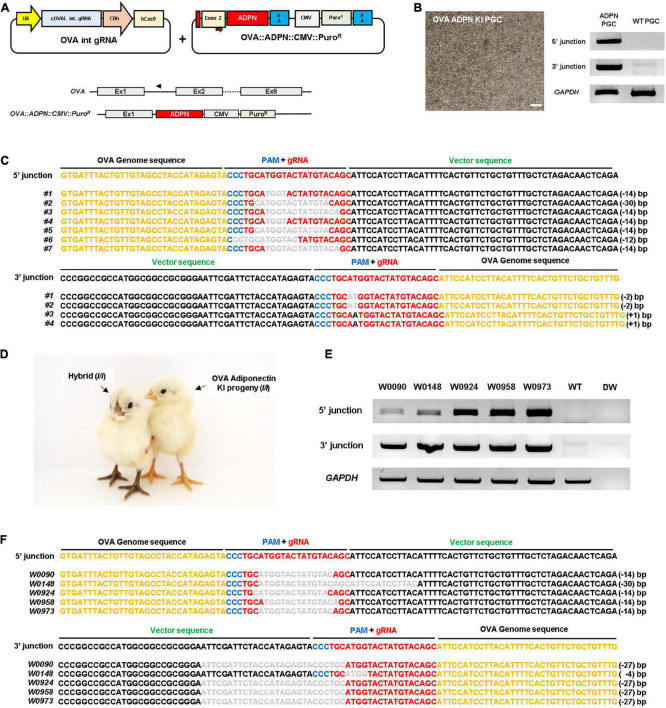
Targeted gene insertion of human *ADPN* at *Ovalbumin (OVA)* gene locus of chicken PGCs and establishment of transgenic chickens. **(A)** A schematic illustration of the *OVA* targeting single guide RNA expressing vector with *ADPN* containing donor plasmid by using non-homologous end joining (NHEJ) repair system. The *OVA* targeting single gRNA expression vector targets the intron region between exon1 and exon2 of *OVA* gene (PAM sequence is indicated by blue bar, guide RNA recognition region is indicated by red bar). The donor plasmid contains *ADPN* fusion DNA including the start codon region of *OVA* exon2 and contains *CMV Puro*^R^** (puromycin resistant gene) cassette. **(B)** *OVA ADPN* KI PGCs and the genomic DNA PCR for *ADPN* gene integration. The modified gene includes the synthesized exon2 partial region, *ADPN* gene, and *CMV Puro*^R^**, and verified the inserted DNA is 5′ junction with OV seq F and ADPN 5′R, and 3′ junction with ADPN 3′F and OV int seq R primers. Scale bar = 200 μm. **(C)** Sequencing analysis of genomic DNA PCR of donor cassette gene insertion in chicken PGCs using 5′ junction and 3′ junction sequence-specific PCR primers. **(D)** Images of donor PGC-derived *OVA ADPN* KI transgenic chicks (*I/I*) and hybrid individuals (*I/i*). **(E)** Genomic DNA PCR of *OVA ADPN* KI chickens using 5′ junction and 3′ junction sequence-specific PCR primers, and **(F)** sequencing analysis of *OVA ADPN* KI chickens. **(C,F)** The black text is the *OVA* genome sequence, the gRNA sequence is red text, the PAM sequence is blue text, and the donor vector sequence is green text. The deleted nucleotide sequence is expressed as a bar in gray uppercase text, substituted nucleotide sequence as gray uppercase text without bar, and inserted nucleotide as gray lowercase.

To produce *ADPN* KI chickens, we first designed a single guide RNA (gRNA) targeting the first *OVA* intron (OVA int gRNA) with a Cas9 expression cassette and donor plasmid containing the human *ADPN* expression cassette, including the partial sequence of exon 2 with the start codon of the *OVA* gene and the recognition site of gRNA to induce NHEJ-mediated knock-in (KI) system (Figure 1A). After transfection of these plasmids into White Leghorn (WL, *I/I*) chicken PGCs, puromycin selection was performed for 24 h to select genome-edited PGCs. The targeted gene insert pattern was verified by genomic DNA PCR using junction-specific primers specific for the 5′ and 3′ ends of the inserted cassette (Figure 1B). DNA sequencing showed that targeted gene insertion into the *OVA* locus caused indel mutations because it contains a gRNA recognition region on the donor plasmid, the so-called bait sequence (Figures 1A, C). Subsequently, the *OVA ADPN* PGCs were transferred into the dorsal aorta of Korean Ogye (KO, *i/i*) recipient embryos at Hamburger and Hamilton (HH) stage 14 − 17, and *OVA ADPN* KI progeny were produced through testcross with WL (*I/I*) hens (Figure 1D). Among the three germline chimeras (S0431, S0433, and S0439), progeny from donor PGCs were generated with efficiencies of 23.81–45.38% (Table 1). Genomic DNA PCR amplification of the 5′ and 3′ junction region confirmed that 18.18 − 23.72% of donor PGC-derived chicks were genome-edited progeny (Figure 1E and Table 1). DNA sequencing of PCR amplicons verified the gene integration patterns of the 5′ and 3′ junctions, confirming that genome-edited chick production was successful through NHEJ-mediated targeted gene insertion into the *OVA* locus (Figure 1F).

**TABLE 1:** Efficiency of germ-line transmission and genome-edited chick production of cOVA ADPN donor PGCs.

Wing tag ID	Donor cell type	No. of hatched chicks	No. of endogenous germ-cell–derived chicks (%)*	No. of donor germ-cell–derived chicks (%)^†^	No. of OVA ADPN KI chicks (%)^‡^
S 0431	*OVA ADPN* WL male PGC	59	37 (62.71)	22 (37.28)	4 (18.18)
S 0433	*OVA ADPN* WL male PGC	130	71 (54.61)	59 (45.38)	14 (23.72)
S 0439	*OVA ADPN* WL male PGC	21	16 (76.19)	5 (23.81)	1 (20.0)

*Test-cross analysis was conducted by mating between WL (*I/I*) and germ-line chimeric KO (*I/i*); that is, transplanted cOVA ADPN knock-in donor PGCs of WL (*I/I*). The phenotype of endogenous recipient germ-cell–derived chick is KO and WL hybrid (*I/i*).

^†^The phenotype of offspring derived from donor PGCs of WL (*I/I*).

^‡^The percentage of *OVA ADPN KI* chicks in donor germ-cell–derived chicks.

### Magnum-specific expression of human ADPN and characteristics of eggs from *OVA ADPN* KI hens

**FIGURE 2:**
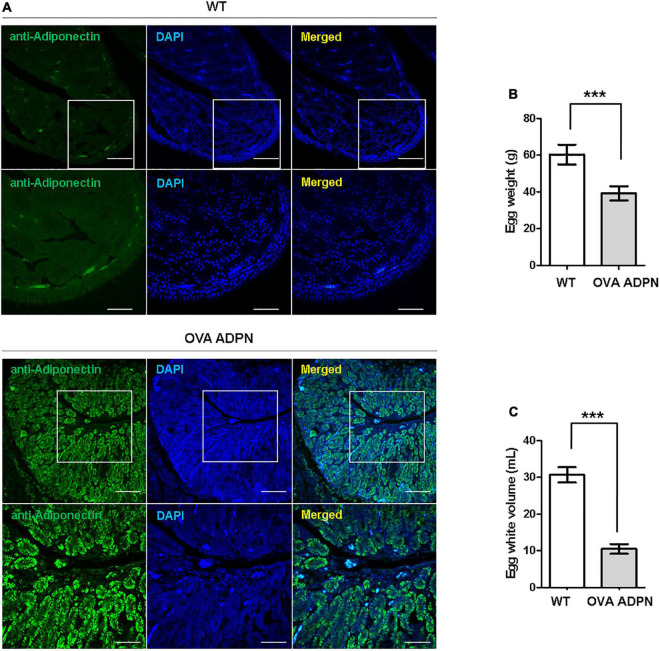
Characterization of G_1_ *OVA ADPN* KI hen’s oviducts and eggs. **(A)** Immunohistochemistry of human adiponectin in magnum part of *OVA ADPN* KI G_1_ and wild type (WT) hen. Scale bars = upper panel: 100 μm, lower panel: 50 μm. **(B)** Comparison of *OVA ADPN* KI chicken egg and WT egg for egg weight (*n* = 21 in WT, 20 in *OVA ADPN* KI). ***P < 0.001. **(C)** Comparison of the egg white properties of G_1_ *OVA ADPN* hen eggs with WT egg white volume (*n* = 15 in WT, 17 in *OVA ADPN* KI). ***P < 0.001.

Following sexual maturation of G_1_ progeny in which the human ADPN gene was inserted into the *OVA* locus, G_2_ progeny were produced. In the case of males, progenies were successfully produced from five G_1_ paternal lines (0011, 0091, 0099, 0198, and 0217), and the production efficiency of *OVA ADPN* KI chicks was 48.91–66.21%. For females, there was not a single offspring produced from four G_1_ *OVA ADPN* KI hens (0090, 0108, 0141, and 0148) (Table 2). The expression pattern of human ADPN was confirmed in the magnum part of the *OVA ADPN* KI hen oviduct, from which OVA protein is known to be secreted. Human ADPN was secreted strongly into the oviductal magnum of egg-laying G_1_ *OVA ADPN* KI hens (Figure 2A). Eggs laid by G_1_ *OVA ADPN* KI hens were lighter than those laid by wild-type eggs of similar age (Figure 2B). In addition, the egg white volume of *OVA ADPN* KI hens was 70% lower than that of wild-type hens (Figure 2C). Overall, *OVA ADPN* genome-edited chickens produced abundant exogenous protein, while cessation of embryo development and hatching of *OVA*-targeted genome-edited hens, and the phenotypic characteristics of *OVA* genome-edited eggs, were similar to those reported in previous studies (18). Therefore, all subsequent experiments were performed on heterozygous (*OVA^ADPN^*^±^) KI females and their eggs.

**TABLE 2:** Production efficiency of G_2_ *OVA ADPN* KI chickens after testcrossing of the G_1_ cOVA ADPN KI chicken with wild-type chickens.

*OVA ADPN* KI chicken ID	Sex	No. of incubated eggs	No. of hatched chicks (%)^†^	No. of *OVA ADPN* KI chicks (%)^‡^
0011	Male	92	74 (80.43)	49 (66.21)
0091	Male	103	77 (74.75)	41 (53.24)
0099	Male	169	129 (76.33)	73 (56.58)
0198	Male	134	124 (92.53)	70 (56.45)
0217	Male	125	92 (73.6)	45 (48.91)
0108	Female	184	0 (0.0)	0 (0.0)
0090	Female	61	0 (0.0)	0 (0.0)
0108	Female	184	0 (0.0)	0 (0.0)
0141	Female	43	0 (0.0)	0 (0.0)
0148	Female	51	0 (0.0)	0 (0.0)

^†^The percentage of the number of chicks hatched after incubation.

^‡^The percentage of *OVA ADPN* KI chicks in hatched chicks.

### Compositional characteristics of human ADPN from *OVA ADPN* KI hens

**FIGURE 3:**
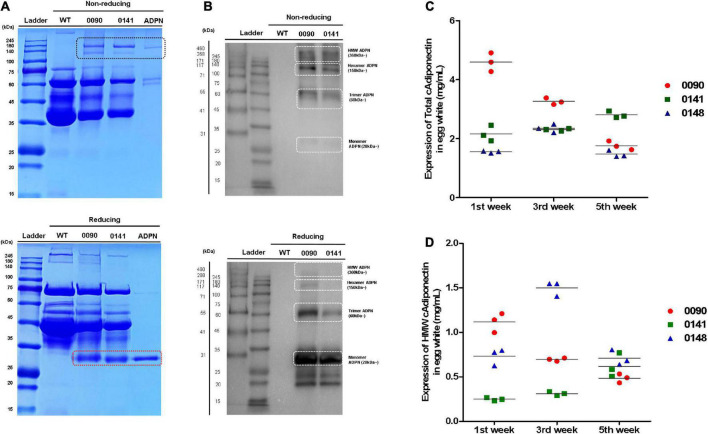
Measurement of total ADPN and HMW ADPN contents in G_1_ *OVA ADPN* KI hen’s eggs. **(A)** Detection of ADPN protein in *OVA ADPN* KI chicken egg white (W0090 and W0141, respectively) by SDS-PAGE under non-reducing and reducing conditions. The black box indicates multimeric adiponectin in non-reducing state. Red box indicate monomer size of ADPN. **(B)** Expression analysis of ADPN protein of *OVA ADPN* KI chicken egg white by western blotting under non-reducing and reducing conditions. White boxes indicate monomer (approximately 28 kDa), trimer (approximately 60 kDa), hexamer (approximately 150 kDa), and high molecular weight form (360 kDa∼), respectively. **(C)** Analysis of total ADPN protein expression level from *OVA ADPN* KI chicken egg (W0090, W0141, and W0148) by ELISA at 1st week, 3rd week, and 5th-week consecutively. **(D)** Analysis of HMW ADPN protein expression level from *OVA ADPN* KI chicken egg (W0090, W0141, and W0148) by ELISA at 1st week, 3rd week, and 5th-week consecutively (*n* = 3, each hen).

We measured human ADPN protein levels in egg white of *OVA ADPN* KI chickens. SDS-PAGE separation of egg whites from heterozygous KI hens 0090 and 0141 was performed together with recombinant ADPN under non-reducing conditions, and bands that were absent from wild-type egg extracts were observed at ≥150 kDa. However, a protein with a molecular weight of 28 kDa, the known molecular weight of human ADPN, was not observed, even though a band of this size was observed under reducing conditions (Figure 3A). Under non-reducing conditions, the monomer ADPN appeared as a smear (KI hens 0090 and 0141). By contrast, trimeric, hexameric, and HMW adiponectin were clearly observed in *OVA ADPN* egg white samples (Figure 3B). In addition, under reducing conditions, the band corresponding to multimeric ADPN was decreased, and the band corresponding to the monomer was increased (Figure 3B). This indicates that in *OVA ADPN* egg white, the multimeric form of ADPN, tends to predominate over the monomeric form.

Subsequently, quantitative analysis was performed on egg white samples from three different *OVA ADPN* KI hens (0090, 0141, and 0148) at 2 weeks intervals for 5 weeks (weeks 1, 3, and 5 after initiation of laying). Expression levels of total ADPN in OVA ADPN eggs ranged from 1.47 to 4.59 mg/mL, and the average was 2.47 mg/mL. The 0090 sample showed a significant difference by each weeks compared to other subjects (0141 and 0148). Among the samples, the expression of ADPN in sample 0090 showed large differences over the 5-week period (±1.4 mg/mL), whereas the expression of ADPN in the other two samples varied little (±0.33 mg/mL for 0141 and ±0.48 mg/mL for 0148) (Figure 3C). HMW ADPN levels in OVA ADPN eggs ranged from 0.24 to 1.49 mg/mL, and the average was 0.71 mg/mL (Figure 3D). This suggests that, on average, approximately 29% of HMW ADPN was present in the total ADPN of OVA ADPN chickens’ eggs. Similar to total ADPN expression, the amount of HMW ADPN in eggs in sample 0090 tended to decrease over time. By contrast, the amount of HMW ADPN increased slightly in 0141, and levels in 0148 fluctuated but remained above a certain level. Overall, human ADPN expression levels were high following OVA-targeted gene insertion, and a large proportion of ADPN tended to be multimeric.

### Purification of ADPN protein from *OVA ADPN* egg white

**FIGURE 4:**
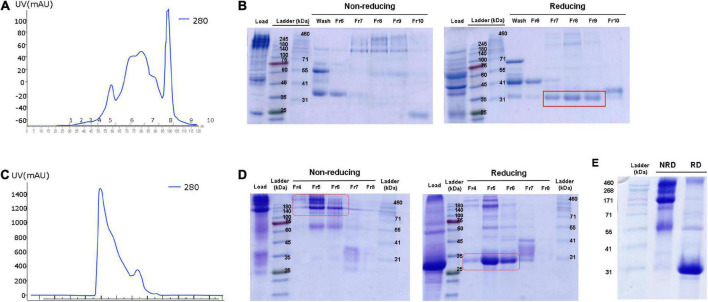
Purification of ADPN from *OVA ADPN* KI chicken egg white. **(A)** Elution profile observed at 280 nm of the purification of ADPN by Hitrap Q hp column. **(B)** Non-reducing and reducing SDS-PAGE gel after Hitrap Q hp column purification. **(C)** Elution profile observed at 280 nm of the purification of ADPN by Superdex G75 analytical size exclusion chromatography. **(D)** Analysis of the ADPN on 10% SDS-PAGE gel following purification by size exclusion chromatography. **(E)** The purified ADPN was loaded on the 10% SDS-PAGE gel. NRD, non-reducing condition; RD, reducing condition.

To purify human ADPN from *OVA ADPN* egg white, we first conducted anion-exchange chromatography as a relatively crude purification step, and several peaks were observed (Figure 4A). SDS-PAGE revealed bands of 140 kDa or higher in fractions 7−9 under non-reducing conditions, but under reducing conditions, these bands were shifted to ∼30 kDa, corresponding to the ADPN monomer (Figure 4B). Subsequently, filtered samples of fractions 7 − 9 were more stringently purified by size exclusion chromatography (SEC) (Figure 4C), which resulted in a single peak. SDS-PAGE of the proteins in this peak revealed a band of ≥140 kDa under non-reducing conditions and a band of ∼30 kDa under reducing conditions (Figure 4D). SDS-PAGE analysis of the concentrated protein isolated by SEC under non-reducing conditions revealed a size of ≥171 kDa, and most proteins were ∼30 kDa under reducing conditions (Figure 4E). In conclusion, these results reveal that most OVA ADPN KI egg-derived human ADPN oligomerized to form a hexamer (≥150 kDa) or HMW (≥360 kDa or more) oligomers, and that human ADPN could be successfully purified from egg white.

### Activation of AMPK and peroxisome proliferator-activated receptor (PPAR)α signaling pathways in C2C12 myoblasts by purified ADPN

**FIGURE 5:**
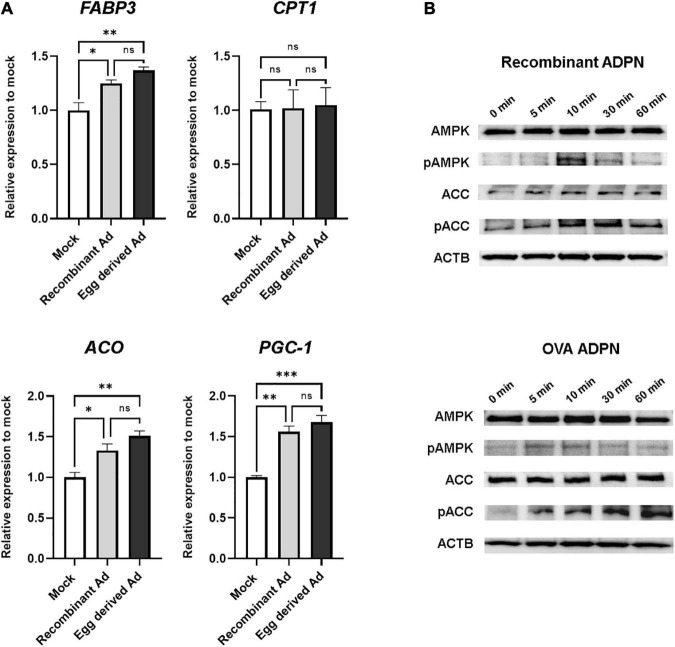
Egg-derived ADPN stimulates PPARα target genes and AMPK signaling pathway. **(A)** The relative mRNA expression levels of PPARα target genes (including *FABP3*, *CPT1*, *ACO*, and *PGC-1*) after 3 h treatment of 2 μg/ml adiponectin on C2C12 myoblast cell line. The amount of mRNA was normalized to *GAPDH*. To determine if any significant differences existed between mock control group, recombinant Ad group, and egg derived Ad group in PPARα target gene expression are validated One-way Anova [Tukeys’ multiple comparison tests (*n* = 3)]. Mock, PBS treatment group; Ad, adiponectin. **(B)** Activation of AMPK pathway by 5 μg/ml of adiponectin treatment. Time-dependent activation of AMPK and ACC by adiponectin. Recombinant Ad (ADPN), commercially available recombinant human adiponectin; Egg-derived Ad (ADPN), purified adiponectin from *OVA ADPN* KI chicken egg. ACTB (Beta–actin) was used as a loading control. Significant differences between groups are indicated as *P < 0.05, ^**^P < 0.01, and ^***^P < 0.001.

Since one of the key functions of ADPN is fatty acid oxidation mediated by AMPK and (PPAR)α in muscle cells, we next investigated the intracellular activity of purified ADPN *in vitro*. When purified ADPN was used to treat the C2C12 myoblast cell line, expression levels of target genes of PPARα including *FABP3*, *ACO*, and *PGC-1* were significantly increased (Figure 5A). Although the difference was not significant, upregulation of these genes tended to be higher in the *OVA ADPN* treatment group than in the recombinant ADPN group. However, there was no significant increase in expression of *CPT1*, a known target gene.

To investigate regulation of AMPK signaling by purified ADPN, western blotting was performed to assess phosphorylation of the target protein in a time-dependent manner. Similar to the recombinant ADPN-treated group, phosphorylation of AMPK by purified ADPN was increased at 10 min, and phosphorylation of acetyl-CoA carboxylase (ACC), a signaling molecule acting downstream of AMPK, was increased from 10 to 60 min (Figure 5B). Overall, induction of AMPK and PPARα activity in muscle cells of *OVA ADPN* KI chicken egg-derived ADPN was similar to that observed for recombinant ADPN, highlighting it as a candidate drug for metabolic diseases such as T2D.

## Discussion

Here, we describe the production of *OVA*-targeted genome-edited chickens that accumulate human ADPN in egg white, and the purified protein has potential for treating chronic metabolic diseases such as T2D and obesity. We successfully produced functional human ADPN in predominantly multimeric forms and showed that purified human ADPN from egg white can promote fatty acid oxidation pathways *in vitro*.

In present study, we showed that *OVA ADPN* chickens can be used to express strongly human ADPN in the oviduct and secrete it into egg white. The egg white of eggs from *OVA ADPN* chickens was low in volume and opaque, consistent with a previous report showing that human interferon beta was accumulated in egg white upon *OVA* targeting (18). In both studies, the target proteins accumulated at high concentrations (>1 mg/mL) in egg white. Also, because of the high expression level of OVA protein that accounts for 50% of egg white, disruption of one OVA allele caused a reduction in expression and quantity of OVA protein by half in the heterozygous knock-in animal. This phenomenon was indirectly observed in our SDS-PAGE results (Figure 3A); the intensity of OVA bands was reduced in *OVA ADPN* egg white samples compared with wild-type samples. Furthermore, SDS-PAGE revealed the amounts of other major egg white proteins in *OVA ADPN* egg white samples were reduced by *ADPN* expression or the reduction in *OVA* expression. This indicates that the targeted gene insertion into the OVA gene locus may affect the secretion of egg white proteins into egg white and reduce egg white volume and weight in *OVA ADPN* eggs. Similarly, in the previous study, the expression of the exogenous protein in OVA-edited eggs showed a decrease in egg size (18). Therefore, we speculated that the egg white protein composition changes may affect egg white shape, volume, and weight in *OVA*-targeted chickens.

Our SDS-PAGE and western blotting results showed that human ADPN predominantly accumulated as multimeric forms, and monomeric ADPN was barely detected in egg white. This indicates that a chicken bioreactor can serve as an efficient production platform for multimeric recombinant human ADPN. The efficient production of multimeric ADPN in chicken may reflect the natural physiological characteristics of chickens. It was reported that high environmental glucose levels can promote multimerization of ADPN (16). Chickens exhibit naturally hyperglycemic characteristics and insulin resistance, which may promote the synthesis and assembly of human ADPN in multimeric form (15).

In obesity, ADPN expression levels are significantly reduced by epigenetic modulation, and the HMW form of ADPN is particularly diminished in serum of T2D patients with obesity (22, 23). Also, *ADPN*^±^ mice possess lower levels of HMW ADPN in serum (20). These results indicate that a reduction in ADPN expression may hinder its multimerization, while high expression levels are beneficial for multimerization. In egg white from *OVA ADPN* chickens, the average concentration of total ADPN ranged from 1.47 to 4.59 mg/mL, hence human ADPN was strongly expressed by the endogenous *OVA* promoter. This strong expression of ADPN in oviduct tubular gland cells may contribute to efficient multimerization of ADPN. During the multimerization process, HMW ADPN, considered the most bioactive form of ADPN, was successfully synthesized and accumulated in egg white, reaching levels of 0.24–1.49 mg/mL and accounting for ∼30% of total ADPN in egg white. Therefore, a chicken bioreactor system can serve as an efficient production platform for generating the most bioactive form of recombinant human ADPN.

Adiponectin stimulates fatty acid oxidation by inducing AMPK and PPARα signaling pathways in muscles and reduces levels of triacylglycerol (TG), which can increase insulin sensitivity (6, 7). These characteristics make ADPN a target protein for the treatment of metabolic diseases. However, the absence of large-scale method for the production of full-length human ADPN with proper bioactivity has prevented using ADPN as a therapeutic agent (13). In the present study, we observed that ADPN purified from egg white stimulated AMPK and ACC phosphorylation in C2C12 myoblast cells, as described previously for recombinant ADPN derived from insect cells. Furthermore, ADPN purified from egg white also stimulated PPARα signaling pathways and induced the expression of PPARα target genes including *FABP3*, *ACO*, and *PGC-1* except for *CPT1* in C2C12 myoblast cells, to a similar extent as recombinant ADPN. Although *CPT1* expression was not changed by egg-derived ADPN in our qRT-PCR analysis, it was not verified in a dose-dependent and time-dependent manner of ADPN treatment. Therefore, presumably, if the experimental conditions are different, the expression level of *CPT1* could be confirmed differently. These results indicate that full-length recombinant human ADPN with functional activity for the induction of AMPK and PPARα signaling pathways could be produced on a large-scale from egg white, and that the chicken bioreactor system could provide an economical source of recombinant human ADPN for the treatment of metabolic diseases.

In the present study, we focused on the efficient production of human ADPN in the egg white of genome-edited chickens and we have not yet verified the *in vivo* efficacy and pharmacokinetics of egg white-derived ADPN. Further studies will be required to verify half-life and fatty acid metabolism regulation *in vivo* in near future.

Collectively, our results demonstrated that a functional human ADPN can be efficiently produced using a chicken egg bioreactor system. Although ADPN has potential to be developed as a biopharmaceutical for various metabolic diseases, its relatively high serum concentration, and complex multimeric structure have been major bottlenecks hampering its development as a cost-effective therapeutic agent. Our findings indicate that a chicken egg bioreactor system can overcome these bottlenecks, and thereby contribute to the future development of recombinant ADPN as an effective therapeutic agent for various metabolic diseases.

## Data availability statement

The datasets presented in this study can be found in online repositories. The names of the repository/repositories and accession number(s) can be found in the article/Supplementary material.

## Ethics statement

All experimental procedures and care of chickens was approved by the Institute of Laboratory Animal Resources, Seoul National University and all methods were carried out in accordance with ARRIVE (Animal Research: Reporting of *In Vivo* Experiments) guidelines and approved by the Institutional Animal Care and Use Committee (IACUC, SNU-220311-1) of Seoul National University, Korea.

## Author contributions

JH conceived and supervised the study. YK and JP designed the research, carried out and analyzed experiments, and wrote the draft of the manuscript. HC and KJ conducted protein purification and analysis from OVA ADPN KI hen’s egg white. KL and JS analyzed the function of purified ADPN and KP assisted in genome-edited animal management. All authors read and approved the final manuscript.

## Footnotes

1
http://blast.ncbi.nlm.nih.gov
