# Supplementary material for: Efficient production of recombinant human adiponectin in egg white using genome edited chickens
Source: Front Nutr. 2023 Jan 24;9:1068558. doi: 10.3389/fnut.2022.1068558 (PMC9902655; doi:10.3389/fnut.2022.1068558)
Supplement: Supplementary file 1 [file Table_1.DOCX]

**Supplementary table S1. Oligonucleotide sequences used in this study.**

| ID | Sequence (5’ to 3’) | Usage |
| --- | --- | --- |
| *OVA* intron #2 F | **CACCTGCATGGTACTATGTACAGC** | ***OVA* intron targeting CRISPR/Cas9 vector construction** |
| *OVA* intron #2 R | **AAACGCTGTACATAGTACCATGCAC** | ***OVA* intron targeting CRISPR/Cas9 vector construction** |
| *OVA* seq F | **GCAACTGGCTTCTGGGACA** | **DNA PCR and genome sequencing** |
| ADPN 5’ R | **TCTCTCCCATCTCTCCCGGG** | **DNA PCR and genome sequencing** |
| ADPN 3’ F | **TCTTCGGGGCGAAAACTCTC** | **DNA PCR and genome sequencing** |
| *OVA* int seq R | **AAGTGCTGTGGCTCCATTGA** | **DNA PCR and genome sequencing** |
| Mouse *FABP3* F | **CCCCTCAGCTCAGCACCAT** | **quantitative RT-PCR** |
| Mouse *FABP3* R | **CAGAAAAATCCCAACCCAAGAAT** | **quantitative RT-PCR** |
| Mouse *CPT1* F | **GGCATCATCACTGGTGTGTTC** | **quantitative RT-PCR** |
| Mouse *CPT1* R | **TGGTGTCTAGGGTCCGATTG** | **quantitative RT-PCR** |
| Mouse *ACO* F | **GGGCATCCTGAGCCTTTGGA** | **quantitative RT-PCR** |
| Mouse *ACO* R | **TGACCCATCTCTGTCTGCGC** | **quantitative RT-PCR** |
| Mouse *PGC-1* F | **AGCCGTGACCACTGACAACGAG** | **quantitative RT-PCR** |
| Mouse *PGC-1* R | **GCTGCATGGTTCTGAGTGCTAAG** | **quantitative RT-PCR** |
| Mouse *GAPDH* F | **CAGAACATCATCCCTGCATCC** | **quantitative RT-PCR** |
| Mouse *GAPDH* R | **CAGATGCCTGCTTCACCACC** | **quantitative RT-PCR** |
